# In-Hospital vs 30-Day Sepsis Mortality at US Safety-Net and Non–Safety-Net Hospitals

**DOI:** 10.1001/jamanetworkopen.2024.12873

**Published:** 2024-05-31

**Authors:** Anica C. Law, Nicholas A. Bosch, Yang Song, Archana Tale, Karen E. Lasser, Allan J. Walkey

**Affiliations:** 1 The Pulmonary Center, Department of Medicine, Boston University School of Medicine, Boston, Massachusetts; 2 Richard A and Susan F Smith Center for Outcomes Research in Cardiology, Beth Israel Deaconess Medical Center, Boston, Massachusetts; 3 Section of General Internal Medicine, Boston Medical Center, Boston, Massachusetts; 4 Division of Health Systems Science, Department of Medicine, University of Massachusetts Chan Medical School, Worcester

## Abstract

**Importance:**

In-hospital mortality of patients with sepsis is frequently measured for benchmarking, both by researchers and policymakers. Prior studies have reported higher in-hospital mortality among patients with sepsis at safety-net hospitals compared with non–safety-net hospitals; however, in critically ill patients, in-hospital mortality rates are known to be associated with hospital discharge practices, which may differ between safety-net hospitals and non–safety-net hospitals.

**Objective:**

To assess how admission to safety-net hospitals is associated with 2 metrics of short-term mortality (in-hospital mortality and 30-day mortality) and discharge practices among patients with sepsis.

**Design, Setting, and Participants:**

Retrospective, national cohort study of Medicare fee-for-service beneficiaries aged 66 years and older, admitted with sepsis to an intensive care unit from January 2011 to December 2019 based on information from the Medicare Provider Analysis and Review File. Data were analyzed from October 2022 to September 2023.

**Exposure:**

Admission to a safety-net hospital (hospitals with a Medicare disproportionate share index in the top quartile per US region).

**Main Outcomes and Measures:**

Coprimary outcomes: in-hospital mortality and 30-day mortality. Secondary outcomes: (1) in-hospital do-not-resuscitate orders, (2) in-hospital palliative care delivery, (3) discharge to a postacute facility (skilled nursing facility, inpatient rehabilitation facility, or long-term acute care hospital), and (4) discharge to hospice.

**Results:**

Between 2011 and 2019, 2 551 743 patients with sepsis (mean [SD] age, 78.8 [8.2] years; 1 324 109 [51.9%] female; 262 496 [10.3%] Black, 2 137 493 [83.8%] White, and 151 754 [5.9%] other) were admitted to 666 safety-net hospitals and 1924 non–safety-net hospitals. Admission to safety-net hospitals was associated with higher in-hospital mortality (odds ratio [OR], 1.09; 95% CI, 1.06-1.13) but not 30-day mortality (OR, 1.01; 95% CI, 0.99-1.04). Admission to safety-net hospitals was associated with lower do-not-resuscitate rates (OR, 0.86; 95% CI, 0.81-0.91), palliative care delivery rates (OR, 0.66; 95% CI, 0.60-0.73), and hospice discharge (OR, 0.82; 95% CI, 0.78-0.87) but not with discharge to postacute facilities (OR, 0.98; 95% CI, 0.95-1.01).

**Conclusions and Relevance:**

In this cohort study, among patients with sepsis, admission to safety-net hospitals was associated with higher in-hospital mortality but not with 30-day mortality. Differences in in-hospital mortality may partially be explained by greater use of hospice at non–safety-net hospitals, which shifts attribution of death from the index hospitalization to hospice. Future investigations and publicly reported quality measures should consider time-delimited rather than hospital-delimited measures of short-term mortality to avoid undue penalty to safety-net hospitals with similar short-term mortality.

## Key Points

**Question:**

Among patients with sepsis, is admission to a safety-net hospital associated with increased 30-day mortality and differences in discharge practices?

**Findings:**

In this cohort study of more than 2 million patients with sepsis, admission to safety-net hospitals was associated with higher in-hospital mortality, but not 30-day mortality. Admission to safety-net hospitals was associated with decreased discharge to hospice.

**Meaning:**

These findings suggest in-hospital mortality rates are associated with differences in discharge practices (eg, by shifting attribution of death from the index hospitalization to hospice), raising concern that current sepsis quality measures using in-hospital mortality could unfairly penalize safety-net hospitals.

## Teaser

This cohort study evaluates how admission to safety-net hospitals is associated with in-hospital mortality, 30-day mortality, and discharge practices among patients with sepsis.

## Introduction

Sepsis is a leading cause of death and disability and a key target of state and federal quality measures. In 2013, New York legislated statewide sepsis regulations, mandating implementation of sepsis protocols and reporting of risk-adjusted in-hospital mortality.^1^ Hospitals with lower (or higher) than expected in-hospital mortality are publicly identified as high (or low) performers. Other states^2^ and the Centers for Medicare and Medicaid Services (CMS) have since legislated similar process measures,^3^ and CMS is planning an additional sepsis outcome measure.^3^

However, public outcome reporting has the potential to exacerbate disparities. Safety-net hospitals (ie, hospitals that care for a disproportionately high share of low-income and underinsured patients) have been reported to have higher risk-adjusted in-hospital mortality among patients with sepsis.^4,5,6^ Safety-net hospitals face unique challenges, including fewer resources and narrower operating margins,^7^ as well as a patient population with decreased access to preventative care, leading to more complex disease at patient presentation.^8^ To avoid further penalizing safety-net hospitals, metrics chosen for public reporting must fairly represent patient-centered outcomes. Specifically, in-hospital mortality rates are known to be influenced by other variables, including hospital transfer practices,^9^ which shift the attribution of death from the hospital to other sites. In contrast, time-delimited outcome measures such as 30-day mortality are less dependent on transfer practices. We compared in-hospital and 30-day mortality, as well as discharge patterns, between safety-net hospitals and non–safety-net hospitals among patients admitted with sepsis across the US. We hypothesized that in-hospital mortality—but not 30-day mortality—would be higher for safety-net hospitals as compared with non–safety-net hospitals, along with greater shifting of deaths to the posthospitalization setting by non–safety-net hospitals.

## Methods

Using the 100% Medicare Provider Analysis and Review File, the Master Beneficiary Summary File, the Chronic Conditions Warehouse,^10^ and the 2016 American Hospital Association Annual Survey Database, we identified patients with sepsis aged 66 years or older who were admitted to an intensive care unit between January 1, 2011, and December 31, 2019. Patients with sepsis were identified by a combination of diagnosis codes for infection and acute organ dysfunction,^11^ mirroring prior studies assessing sepsis outcomes at hospitals serving disproportionately racial and ethnic minority and/or underinsured patients.^4^ Safety-net hospitals were defined as hospitals with a Medicare Disproportional Share Index in the top quartile per US region using the CMS Impact File.^12,13^ This study was deemed exempt from review and informed consent by Beth Israel Deaconess Medical Center and Boston University Medical Center institutional review boards because it was deemed non–human participants research. The study was also exempt from the need for informed consent because data were deidentified. This study is reported following the Strengthening the Reporting of Observational Studies in Epidemiology (STROBE) reporting guidelines for observational studies.

### Outcomes

Coprimary outcomes included in-hospital mortality and 30-day mortality. Secondary outcomes included (1) in-hospital do-not-resuscitate (DNR) orders (*International Classification of Diseases*, *Ninth *and *Tenth Revisions [ICD-9 *and* ICD-10]*: *ICD-9* V49.86; *ICD10* Z66), (2) in-hospital palliative care delivery (*ICD-9* V66.7; *ICD10* Z51.5), (3) discharge to a postacute facility (skilled nursing facility, inpatient rehabilitation facility, or long-term acute care hospital) and (4) discharge to hospice (facility or home). Among patients who died within 30 days of admission, we quantified site of death (in-hospital, in-hospice, or other [postacute facility, home, or other]).

### Statistical Analysis

We used multivariable hierarchical regression with safety-net hospital status as our exposure of interest, hospital of admission as a random intercept, and hospital- and patient-level covariables as fixed effects (hospital characteristics: size, region, rural or urban status, teaching status, and profit and ownership; patient characteristics: sex, age, social vulnerability index,^14^ Medicare and Medicaid dual-eligibility, surgical status,^15^ chronic comorbidities [assessed 1-5 years before index admission, per Chronic Conditions Warehouse algorithms],^10^ and acute severity of illness [via algorithm assessing for presence of acute organ dysfunction]). We had previously validated the algorithm assessing for acute organ dysfunctions, with an area under the receiver operating curve (AUC) higher than that of the Sequential Organ Failure Assessment (SOFA) score for estimating hospital mortality (0.80 vs 0.74).^16^ For the current study, we extended the prior validation by assessing the ability to estimate the acute organ dysfunction algorithm at safety-net hospitals and non–safety-net hospitals separately and found similar discrimination (AUC 0.79 at safety-net hospitals; AUC 0.80 at non–safety-net hospitals; AUC for SOFA was 0.72 and 0.76, respectively) (eFigure 1 in Supplement 1). Less than 1% of patients had missing data (social vulnerability index); these patients were dropped in the adjusted analysis. In the primary analysis, we did not adjust for race because although safety-net hospitals are known to serve a higher proportion of racial and ethnic minority patients,^13,17^ we did not suspect a biologic association between race and patient outcomes, outside of previously adjusted differences in socioeconomic status or higher comorbid or acute illness burden. However, as race may be associated with other unmeasured confounders (eg, severity of chronic illness or end-of-life preferences^18,19^), we conducted a sensitivity analysis with adjustment for patient race and ethnicity. Patient race and ethnicity were determined from the Medicare Master Beneficiary Summary File, which derives self-reported race and ethnicity (categorized as Black, White, and other [Asian, Hispanic, North American Native, other]) from the Social Security Administration. We also conducted sensitivity analyses with (1) hospital referral region^20^ as a random effect and (2) a modified Poisson model with a sandwich variance estimator^21,22^ to estimate risk ratios instead of odds ratios. In exploratory analysis, we determined the number of safety-net hospitals that would be considered low-performing outliers (defined as hospitals with a risk-standardized mortality rate that was statistically significantly greater than the mean) for in-hospital mortality vs 30-day mortality; we tested for statistical significance with χ^2^ tests. Statistical testing was 2-tailed, with α = .05 indicating statistical significance, using SAS version 9.4 (SAS Institute).

## Results

Between 2011 and 2019, 2 551 743 eligible patients (mean [SD] age, 78.8 [8.2] years; 1 324 109 [51.9%] female; 262 496 [10.3%] Black, 2 137 493 [83.8%] White, and 151 754 [5.9%] other) with sepsis were admitted to 666 safety-net hospitals and 1924 non–safety-net hospitals (hospital characteristics shown in eTable 1 in Supplement 1). Baseline patient characteristics are shown in the Table; safety-net hospitals had a lower proportion of White patients and higher mean social vulnerability index.

**Table.** Baseline Characteristics of Patients With Sepsis Cared for at Safety-Net and Non–Safety-Net Hospitals

Characteristic	Patients, No. (%)		
	Safety-net hospitals (n = 650 749 patients at 666 hospitals)	Non–safety-net hospitals (n = 1 900 994 patients at 1924 hospitals)	Total (N = 2 551 743 patients)
Age, mean (SD), y	78.6 (8.3)	78.9 (8.2)	78.8 (8.2)
Sex			
Female	337 326 (51.8)	986 783 (51.9)	1 324 109 (51.9)
Male	313 423 (48.2)	914 211 (48.1)	1 227 634 (48.1)
Race			
Black	111 217 (17.1)	151 279 (8.0)	262 496 (10.3)
White	480 221 (73.8)	1 657 272 (87.2)	2 137 493 (83.8)
Other^a^	59 311 (9.1)	92 443 (4.9)	151 754 (5.9)
Social Vulnerability Index, mean (SD)^b^	0.6 (0.3)	0.5 (0.3)	0.5 (0.3)
Medicaid/Medicare dual eligibility	199 813 (30.7)	372 486 (19.6)	572 299 (22.4)
Surgical patient	178 890 (27.5)	486 996 (25.6)	665 886 (26.1)
Acute organ dysfunction			
Cardiovascular	310 077 (47.6)	890 438 (46.8)	1 200 515 (47.0)
Respiratory	403 954 (62.1)	1 121 905 (59.0)	1 525 859 (59.8)
Neurologic	176 135 (27.1)	513 031 (27.0)	689 166 (27.0)
Hematologic	117 272 (18.0)	335 193 (17.6)	452 465 (17.7)
Hepatic	27 756 (4.3)	80 927 (4.3)	108 683 (4.3)
Kidney	394 952 (60.7)	1 129 555 (59.4)	1 524 507 (59.7)
Chronic comorbidities			
Alzheimer disease	96 076 (14.8)	233 319 (12.3)	329 395 (12.9)
Acute myocardial infarction	63 771 (9.8)	178 008 (9.4)	241 779 (9.5)
Anemia	495 169 (76.1)	1 402 600 (73.8)	1 897 769 (74.4)
Asthma	78 958 (12.1)	222 300 (11.7)	301 258 (11.8)
Atrial fibrillation	205 073 (31.5)	658 150 (34.6)	863 223 (33.8)
Bipolar disorder	42 267 (6.5)	105 193 (5.5)	147 460 (5.8)
Breast cancer	30 004 (4.6)	97 588 (5.1)	127 592 (5.0)
Colorectal cancer	28 139 (4.3)	87 048 (4.6)	115 187 (4.5)
Endometrial cancer	6082 (0.9)	17 847 (0.9)	23 929 (0.9)
Lung cancer	35 096 (5.4)	112 250 (5.9)	147 346 (5.8)
Prostate cancer	37 477 (5.8)	118 115 (6.2)	155 592 (6.1)
Leukemias and lymphomas	26 864 (4.1)	87 054 (4.6)	113 918 (4.5)
Congestive heart failure	418 933 (64.4)	1 209 722 (63.6)	1 628 655 (63.8)
Chronic kidney disease	509 945 (78.4)	1 463 862 (77.0)	1 973 807 (77.4)
Chronic obstructive pulmonary disease and bronchiectasis	308 031 (47.3)	910 093 (47.9)	1 218 124 (47.7)
Depressive disorders	231 019 (35.5)	683 857 (36.0)	914 876 (35.9)
Diabetes	345 344 (53.1)	916 011 (48.2)	1 261 355 (49.4)
Hip or pelvic fracture	26 580 (4.1)	81 327 (4.3)	107 907 (4.2)
Hyperlipidemia	431 455 (66.3)	1 290 491 (67.9)	1 721 946 (67.5)
Human immunodeficiency virus and/or acquired immunodeficiency syndrome	3408 (0.5)	4105 (0.2)	7513 (0.3)
Hypertension	597 373 (91.8)	1 727 445 (90.9)	2 324 818 (91.1)
Acquired hypothyroidism	164 218 (25.2)	518 318 (27.3)	682 536 (26.7)
Ischemic heart disease	448 486 (68.9)	1 272 066 (66.9)	1 720 552 (67.4)
Liver disease, cirrhosis, and other liver conditions	102 851 (15.8)	276 021 (14.5)	378 872 (14.8)
Viral hepatitis	20 538 (3.2)	39 627 (2.1)	60 165 (2.4)
Obesity	189 408 (29.1)	548 694 (28.9)	738 102 (28.9)
Osteoporosis	82 307 (12.6)	251 965 (13.3)	334 272 (13.1)
Peripheral vascular disease	288 221 (44.3)	792 913 (41.7)	1 081 134 (42.4)
Pressure and chronic ulcers	175 539 (27.0)	470 773 (24.8)	646 312 (25.3)
Rheumatoid arthritis or osteoarthritis	302 539 (46.5)	878 057 (46.2)	1 180 596 (46.3)
Sickle cell disease	453 (0.1)	770 (<0.1)	1223 (<0.1)
Stroke or transient ischemic attack	126 109 (19.4)	312 874 (16.5)	438 983 (17.2)
Alcohol use disorders	49 679 (7.6)	127 705 (6.7)	177 384 (7.0)
Drug use disorders	42 395 (6.5)	121 395 (6.4)	163 790 (6.4)
Tobacco use	146 667 (22.5)	410 405 (21.6)	557 072 (21.8)
Hospital size			
Small (1-200)	132 175 (20.3)	552 367 (29.1)	684 542 (26.8)
Medium (201-400)	192 529 (29.6)	728 586 (38.3)	921 115 (36.1)
Large (>400)	326 045 (50.1)	620 041 (32.6)	946 086 (37.1)
Hospital ownership			
For profit	130 409 (20.0)	282 394 (14.9)	412 803 (16.2)
Private nonprofit	427 816 (65.7)	1 440 222 (75.8)	1 868 038 (73.2)
Public	92 524 (14.2)	178 378 (9.4)	270 902 (10.6)
Hospital teaching status	508 844 (78.2)	1 262 919 (66.4)	1 771 763 (69.4)
Rural hospital location	14 716 (2.3)	21 046 (1.1)	35 762 (1.4)
Hospital region			
Northeast	111 249 (17.1)	341 151 (17.9)	452 400 (17.7)
Midwest	90 302 (13.9)	307 567 (16.2)	397 869 (15.6)
South	365 571 (56.2)	936 495 (49.3)	1 302 066 (51.0)
West	83 627 (12.9)	315 781 (16.6)	399 408 (15.7)

^a^ Other race includes: Asian, Hispanic, North American Native, and other, as per Medicare Master Beneficiary Summary File, which derives self-reported race and ethnicity from the Social Security Administration.

^b^ Social vulnerability index: each patient’s census tract is based on 15 social factors, including poverty, lack of vehicle access, and crowded housing. A census tract’s index is a percentile ranking and reflects the proportion of tracts in the country that are equal to or lower in terms of social vulnerability.

**Figure 1.**
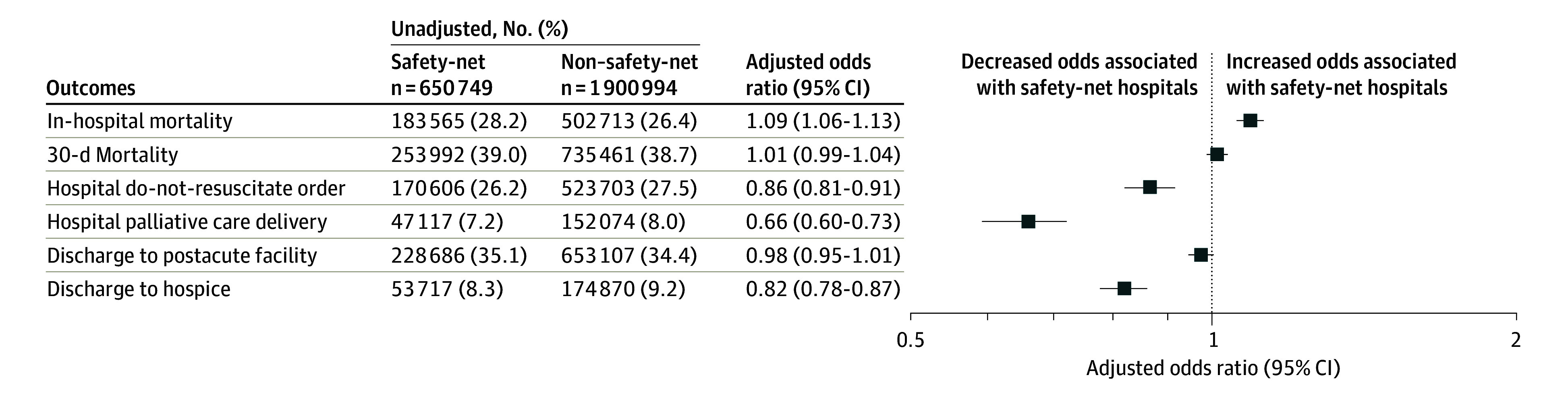
Patient Outcomes Unadjusted outcomes of patients with sepsis are shown on the left. Adjusted odds ratios for the association between admission to safety-net hospitals and patient outcomes are shown on the right. Hierarchical multivariable regression models (with hospital of admission as random effect) are adjusted for all characteristics listed in the Table, except for race.

**Figure 2.**
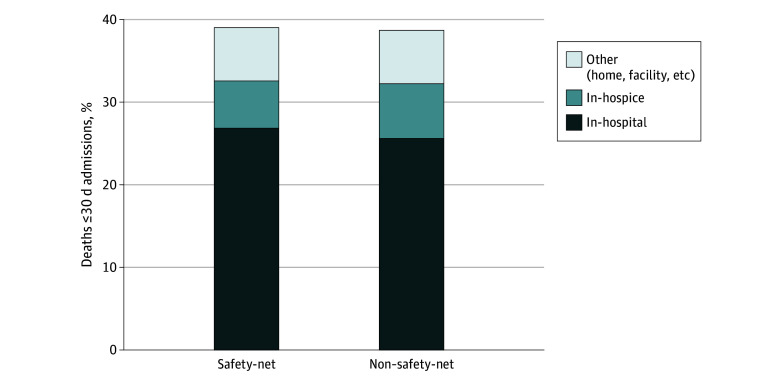
Sites of Death Occurring Within 30 Days, Unadjusted Proportions Among patients who died within 30 days of admission (253 992 [39%] admitted to safety-net hospitals vs 735 461 [39%] admitted to non–safety-net hospitals), deaths attributed to the hospital of admission (dark blue, 174 752 [27%] vs 486 633 [26%]), hospice (medium blue, 37 251 [6%] vs 126 163 [7%]), and other sites (light blue, 41 989 [6%] vs 122 665 [6%]) are shown.

Outcomes of patients at safety-net hospitals and non–safety-net hospitals are shown in Figure 1 (safety–net vs non–safety-net hospitals unadjusted proportions; in-hospital mortality: 183 565 [28.2%] vs 502 713 [26.4%]; 30-day mortality: 253 992 [39.0%] vs 735 461 [38.7%]). Of patients who died within 30 days, site of death is shown in Figure 2 (safety-net vs non–safety-net hospitals unadjusted proportions; in-hospital: 174 752 [26.8%] vs 486 633 [25.6%]; hospice: 37 251 [5.7%] vs 126 163 [6.6%]; other: 41 989 [6.5%] vs 122 665 [6.5%]). In adjusted analysis (Figure 1), admission to safety-net hospitals was associated with higher in-hospital mortality (odds ratio [OR], 1.09; 95% CI, 1.06-1.13) but not 30-day mortality (OR, 1.01; 95% CI, 0.99-1.04). Admission to safety-net hospitals was associated with lower in-hospital DNR rates (OR, 0.86; 95% CI, 0.81-0.91), in-hospital palliative care delivery rates (OR, 0.66; 95% CI, 0.60-0.73), and hospice discharge (OR, 0.82; 95% CI, 0.78-0.87) but not with discharge to postacute facilities (OR, 0.98; 95% CI, 0.95-1.01). Results were similar in sensitivity analyses adjusting for race, using hospital referral region as a random effect, and in modified Poisson models estimating risk ratios (eFigure 2, eTable 2, and eTable 3 in Supplement 1).

In exploratory analysis, 174 out of 666 safety-net hospitals (26.1%) were considered low-performing outliers when ranked by in-hospital mortality. When ranked by 30-day mortality, 88 of the previously ranked low-performing hospitals (51%) were no longer considered low-performing; the total number of hospitals ranked as low-performing decreased to 136 (20.4%; χ^2^_1_ = 121.937; *P* < .001 vs in-hospital mortality).

## Discussion

In this national study of patients with sepsis, admission to safety-net hospitals was associated with higher in-hospital mortality but not 30-day mortality. Differences in in-hospital mortality between safety-net hospitals and non–safety-net hospitals may partially be explained by greater use of hospice at non–safety-net hospitals, which shifts attribution of death from the index hospitalization to hospice. While the magnitude of the differences between safety-net hospitals and non–safety-net hospitals in either in-hospital mortality or hospice discharges were small, the small differences were enough to affect hospital rankings; over half of safety-net hospitals classified as low-performing when ranked by in-hospital mortality were no longer considered low-performing when ranked by 30-day mortality. Our findings can inform the selection of the future sepsis outcome measure proposed by CMS and also raise concern that current sepsis quality measures using in-hospital mortality to rank hospitals (eg, New York’s, which also risk-adjusts using a smaller set of severity-of-illness confounders) could unfairly penalize safety-net hospitals.

Prior studies examining the association of safety-net hospitals with outcomes of patients with sepsis have focused on comparing in-hospital mortality,^4,6^ which is more readily available in large databases, such as the National Inpatient Sample, or adherence to federal process measures (Severe Sepsis/Septic Shock Early Management Bundle [SEP-1]).^4,6^ Our estimates of the increased odds of in-hospital mortality associated with safety-net hospitals were similar to prior studies.^4^ However, we were also able to assess both inpatient and posthospitalization data to demonstrate that 30-day (ie, time-delimited) mortality was not significantly different by safety-net status, despite challenges unique to safety-net hospitals.

The similar 30-day mortality also adds context for prior critiques of the CMS process measure, SEP-1.^4,6^ As SEP-1 has not clearly been shown to be associated with improved mortality,^10,23^ some have raised concerns that the resource-intensive SEP-1 may actually harm safety-net hospitals and widen health disparities as safety-net hospitals struggle to mount the financial and human resources required to comply.^6^ Despite previously-reported lower SEP-1 adherence among safety-net hospitals,^23^ our findings of similar short-term mortality adds to literature suggesting SEP-1 process adherence may not be closely associated with clinical outcomes.^24^ Future studies are needed to more closely assess the association between SEP-1 adherence and 30-day mortality at safety-net hospitals.

Our findings reveal important differences in end-of-life practices between safety-net hospitals and non–safety-net hospitals that may explain differences in in-hospital mortality. Racial minorities^18^ and patients at minority-serving hospitals^25^ receive less palliative care, and palliative care has been shown to increase DNR orders and hospice use.^26,27^ It remains unclear to what degree our observed differences in palliative care receipt and hospice use are culturally and goal-concordant, vs reflective of inadequate implementation of inpatient palliative care (and/or outpatient advanced care planning) at safety-net hospitals—an important area for further study.

### Limitations

Our study has limitations. First, findings among Medicare beneficiaries may not be generalizable to other populations. However, Medicare data allow assessment of posthospitalization mortality and rigorous adjustment for prehospitalization comorbidities. Furthermore, by including only Medicare beneficiaries (by definition, all insured), our study is less confounded by differences in insurance status between populations receiving care at safety-net hospitals and non–safety-net hospitals. Second, cohort inclusion and risk-adjustment by administrative data may be vulnerable to misclassification. However, to place our findings in context of prior work demonstrating inferior outcomes at safety-net hospitals, we defined our cohort by mirroring inclusion criteria for sepsis used in prior studies.^4^ *ICD-10* codes are also commonly used for risk adjustment in state and federal quality metrics.^1,28^ Our claims-based risk adjustment algorithm has been validated against the SOFA score to estimate death in patients with critical illness^16^; our expanded validation of the algorithm for the current study found similar high discrimination at both safety-net and non–safety-net hospitals. While *ICD-10* coding for DNR orders and palliative care consultations are known to have limited sensitivity^29,30^ and may be influenced by differential coding between safety-net and non–safety-net hospitals, the results of the DNR and palliative care analyses are internally consistent with other findings, including reduced discharge to hospice and reduced in-hospice mortality within 30 days at safety-net hospitals.

## Conclusions

In this cohort study of patients with sepsis, admission to safety-net hospitals was associated with higher in-hospital but not 30-day mortality. Differences in in-hospital mortality between safety-net hospitals and non–safety-net hospitals may partially be explained by greater use of hospice at non–safety-net hospitals, which shifts attribution of death from the index hospitalization to hospice. Although in-hospital mortality is often selected as an outcome measure because of its availability in claims databases and hospital medical records without need for posthospitalization follow-up, our findings highlight the importance of examining time-delimited outcomes (eg, 30-day mortality) when hospital discharge patterns differ across important factors such as safety-net status. Our findings can be used to guide selection of outcome measures for publicly reported quality benchmarks to avoid disproportionately penalizing safety-net hospitals.

## Notes

Supplement 1. **eFigure 1. **Performance of Algorithm to Risk-Adjust for Severity of Illness is Similar at Safety-Net and Non–Safety-Net Hospitals **eTable 1. **Characteristics of Safety-Net and Non–Safety-Net Hospitals **eFigure 2.** Patient Outcomes, Adjusted for Race **eTable 2.** Sensitivity Analysis, Using Hospital Referral Region as Random Intercept **eTable 3. **Sensitivity Analysis: Modified Poisson Regression Model to Estimate Risk Ratios

Supplement 2.
Data Sharing Statement
