# Supplementary material for: In-Hospital vs 30-Day Sepsis Mortality at US Safety-Net and Non–Safety-Net Hospitals
Source: JAMA Netw Open. 2024 May 31;7(5):e2412873. doi: 10.1001/jamanetworkopen.2024.12873 (PMC11143462; doi:10.1001/jamanetworkopen.2024.12873)
Supplement: Supplement 1. — eFigure 1. Performance of Algorithm to Risk-Adjust for Severity of Illness is Similar at Safety-Net and Non–Safety-Net Hospitals eTable 1. Characteristics of Safety-Net and Non–Safety-Net Hospitals eFigure 2. Patient Outcomes, Adjusted for Race eTable 2. Sensitivity Analysis, Using Hospital Referral Region as Random Intercept eTable 3. Sensitivity Analysis: Modified Poisson Regression Model to Estimate Risk Ratios [file jamanetwopen-e2412873-s001.pdf]

Supplemental Online Content

Law AC, Bosch NA, Song Y, Tale A, Lasser KE, Walkey AJ. In-hospital vs 30-day sepsis mortality at US safety-net and non–safety-net hospitals. *JAMA Netw Open*. 2024;7(5):e2412873. doi:10.1001/jamanetworkopen.2024.12873

eFigure 1. Performance of Algorithm to Risk-Adjust for Severity of Illness is Similar at Safety-Net and Non–Safety-Net Hospitals

eTable 1. Characteristics of Safety-Net and Non–Safety-Net Hospitals

eFigure 2. Patient Outcomes, Adjusted for Race

eTable 2. Sensitivity Analysis, Using Hospital Referral Region as Random Intercept

eTable 3. Sensitivity Analysis: Modified Poisson Regression Model to Estimate Risk Ratios

This supplemental material has been provided by the authors to give readers additional information about their work.

© 2024 Law AC et al. *JAMA Netw Open.*

eFigure 1. Performance of Algorithm to Risk-Adjust for Severity of Illness is Similar at Safety-Net and Non–Safety-Net Hospitals. In an extension of our previously published validation (using the Premier, Inc, multicenter database, 2016-2020, among adults admitted to the ICU [PMCID: PMC9797032], we validated the algorithm among safety-net and non-safety-net hospitals separately. The receiver operating curves are shown for the claims-based algorithm to predict hospital mortality for (a) safety-net hospitals (area under the receiver operating curve, AUC: 0.79) and (b) non-safety net hospitals (AUC: 0.80). The AUC for the Sequential Organ Failure Assessment (SOFA) was 0.72 and 0.76, respectively.

A. Safety-net hospitals

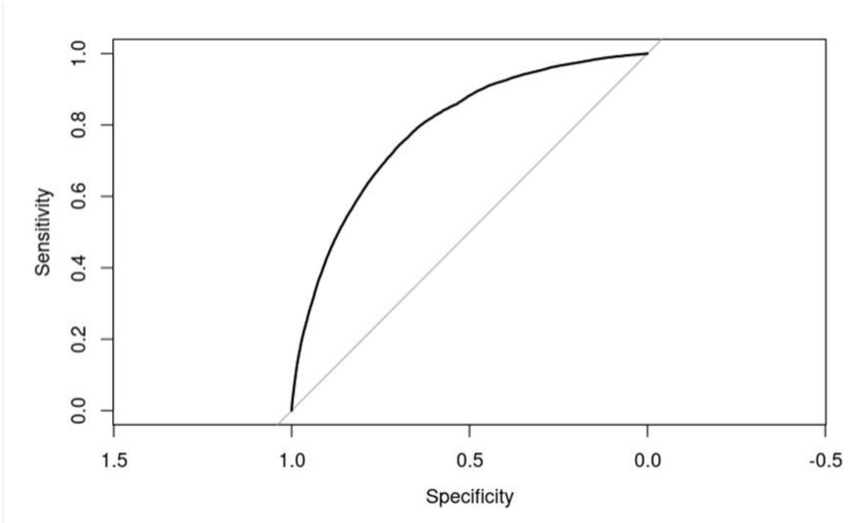

B. Non-safety-net hospitals.

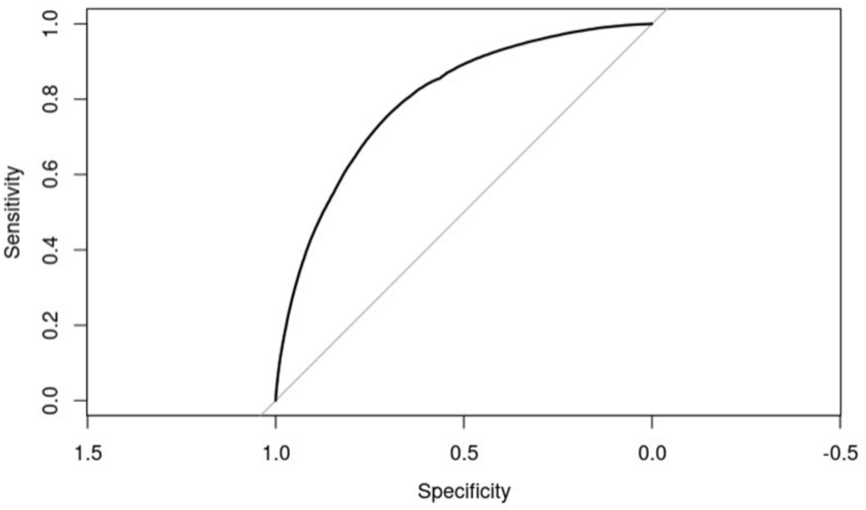

© 2024 Law AC et al. *JAMA Netw Open.*

eTable 1. Characteristics of Safety-Net and Non–Safety-Net Hospitals

**Hospital Characteristics<br>N (%) unless otherwise indicated**	**Safety Net Hospitals<br>(N = 666 hospitals)**	**Non-Safety Net Hospitals<br>(N = 1924 hospitals)**	**Total<br>(N = 2590 hospitals)**
Hospital size			
Small (1 - 200)	301 (45.2%)	1083 (56.3%)	1384 (53.4%)
Medium (201 - 400)	197 (29.6%)	575 (29.9%)	772 (29.8%)
Large (> 400)	168 (25.2%)	266 (13.8%)	434 (16.8%)
Ownership			
For Profit	166 (24.9%)	366 (19.0%)	532 (20.5%)
Private Nonprofit	379 (56.9%)	1347 (70.0%)	1726 (66.6%)
Public	121 (18.2%)	211 (11.0%)	332 (12.8%)
Teaching hospital	408 (61.3%)	967 (50.3%)	1375 (53.1%)
Rural	43 (6.5%)	81 (4.2%)	124 (4.8%)
Hospital region			
Northeast	105 (15.8%)	323 (16.8%)	428 (16.5%)
Midwest	103 (15.5%)	317 (16.5%)	420 (16.2%)
South	337 (50.6%)	893 (46.4%)	1230 (47.5%)
West	121 (18.2%)	391 (20.3%)	512 (19.8%)

© 2024 Law AC et al. *JAMA Netw Open.*

eFigure 2. Patient Outcomes, Adjusted for Race. The adjusted odds ratios for the association between admission to safety-net hospitals and patient outcomes are shown. Models are adjusted for all characteristics listed in Table 1, including Race.

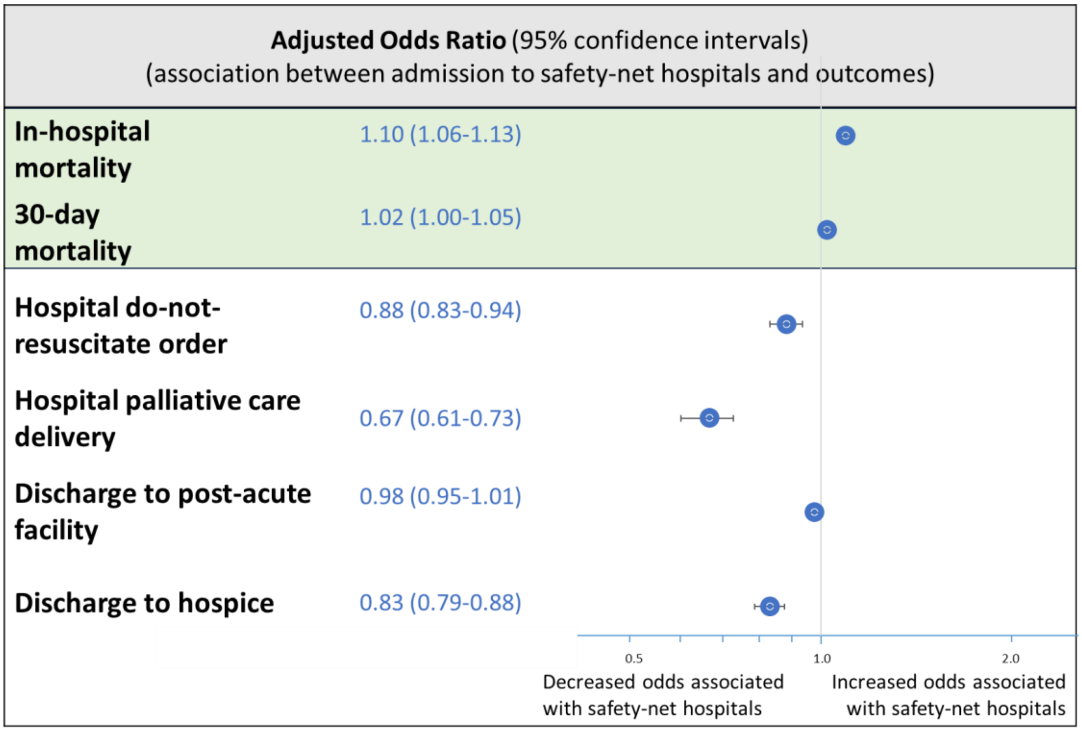

© 2024 Law AC et al. *JAMA Netw Open.*

eTable 2. Sensitivity Analysis, Using Hospital Referral Region as Random Intercept.

Endpoint	Odds Ratio (95% CI)	p-value
In-hospital Mortality	1.05 (1.04, 1.06)	<.0001
30-day Mortality	1.01 (1.01, 1.02)	0.0007
Hospital do-not-resuscitate order	0.94 (0.93, 0.94)	<.0001
Hospital palliative care delivery	0.87 (0.86, 0.88)	<.0001
Discharge to post-acute facility	1.00 (1.00, 1.01)	0.2343
Discharge to hospice	0.91 (0.90, 0.92)	<.0001

© 2024 Law AC et al. *JAMA Netw Open.*

eTable 3. Sensitivity Analysis: Modified Poisson Regression Model to Estimate Risk Ratios.

Endpoint	Risk Ratio (95% CI)	p-value
In-hospital Mortality	1.07 (1.05, 1.09)	<.0001
30-day Mortality	1.01 (1.00, 1.02)	0.0674
Hospital do-not-resuscitate order	0.92 (0.89, 0.94)	<.0001
Hospital palliative care delivery	0.81 (0.75, 0.87)	<.0001
Discharge to post-acute facility	0.97 (0.96, 0.99)	0.0002
Discharge to hospice	0.86 (0.82, 0.89)	<.0001

© 2024 Law AC et al. *JAMA Netw Open.*
