# Supplementary material for: In-Hospital vs 30-Day Sepsis Mortality at US Safety-Net and Non–Safety-Net Hospitals
Source: JAMA Netw Open. 2024 May 31;7(5):e2412873. doi: 10.1001/jamanetworkopen.2024.12873 (PMC11143462; doi:10.1001/jamanetworkopen.2024.12873)
Supplement: Supplement 2. — Data Sharing Statement [file jamanetwopen-e2412873-s002.pdf]

Data Sharing Statement

Law. In-Hospital vs 30-Day Sepsis Mortality at US Safety-Net and Non–Safety-Net Hospitals. *JAMA Netw Open*. Published May 31, 2024. doi:10.1001/jamanetworkopen.2024.12873

Data
Data available: No

Additional Information
Explanation for why data not available: Medicare DUA prohibits sharing
